# Supplementary material for: Deletion of wfs1 Impairs Oligodendrocyte Precursor Cells Dorsal Distribution and Myelination Through the wfs1-hmgcs1 Axis in Zebrafish
Source: Biology (Basel). 2026 Aug 16;15(16):1402. doi: 10.3390/biology15161402 (PMC13510151; doi:10.3390/biology15161402)
Supplement: Supplementary file 1 [file biology-15-01402-s001.zip › File S1-Transcriptome sequencing data and differentially expressed genes (DEGs) in wfs1-- zebrafish at 5 dpf/2.KEGG/wfs1KOvsWT/UP/src/dre00591.html]

dre00591


- dre:406370

- Up regulated genes

406370(7.33922178722892)
- dre:560649

- Up regulated genes

560649(1.22362655394035)

- dre:393592

- Up regulated genes

393592(3.73058515777409)

- dre:553969

- Up regulated genes

553969(1.15424087647056)

- dre:393592

- Up regulated genes

393592(3.73058515777409)

- dre:553969

- Up regulated genes

553969(1.15424087647056)

Close
